# Differential effects of age, cytomegalovirus-seropositivity and end-stage renal disease (ESRD) on circulating T lymphocyte subsets

**DOI:** 10.1186/1742-4933-8-2

**Published:** 2011-01-08

**Authors:** Nicolle HR Litjens, Elly A de Wit, Michiel GH Betjes

**Affiliations:** 1 Department of Internal Medicine, Division of Nephrology, Erasmus Medical Center, Rotterdam, The Netherlands

## Abstract

The age- and cytomegalovirus (CMV)-seropositivity-related changes in subsets and differentiation of circulating T cells were investigated in end-stage renal disease (ESRD) patients (n = 139) and age-matched healthy individuals. The results show that CMV-seropositivity is associated with expansion of both CD4^+ ^and CD8^+ ^memory T cells which is already observed in young healthy individuals. In addition, CMV-seropositive healthy individuals have a more differentiated memory T cell profile. Only CMV-seropositive healthy individuals showed an age-dependent decrease in CD4^+ ^naïve T cells. The age-related decrease in the number of CD8^+ ^naïve T cells was CMV-independent. In contrast, all ESRD patients showed a profound naïve T-cell lymphopenia at every decade. CMV-seropositivity aggravated the contraction of CD4^+ ^naïve T cells and increased the number of differentiated CD4^+ ^and CD8^+ ^memory T cells. In conclusion, CMV-seropositivity markedly alters the homeostasis of circulating T cells in healthy individuals and aggravates the T cell dysregulation observed in ESRD patients.

## Introduction

End-stage renal disease (ESRD) is associated with an immune defect characterized by increased susceptibility for infections and decreased humoral responses to T-cell dependent antigens like HBsAg [1,2]. Circulating T lymphocytes can be dissected into subsets of naïve and memory T lymphocytes using the common leukocyte antigen CD45RO (memory marker) and the chemokine receptor CCR7 which is important for homing of T cells to lymphoid organs. Differential expression of CD45RO and CCR7 allows for further dissection of functionally different T lymphocyte subsets; naïve T cells (Tnaive, expressing CCR7), central-memory T cells (Tcm, expressing CCR7) and effector-memory (Tem, no expression of CCR7) [3]. In addition, within the CD8^+ ^T lymphocyte subset an extra late effector memory population can be distinguished, lacking both CCR7 and CD45RO (Temra) [4,5]. The loss of expression of the co-stimulatory molecule CD28 and increased expression of CD57 on memory T cells is indicative of a later stage of differentiation of T cells with diminished replicative capacity [6].

Progressive loss of renal function is associated with a progressive decrease in the size of the the naïve T cell compartment, a decrease within CD4^+ ^Tcm lymphocytes and a significant increase in CD8^+ ^Temra [7,8]. In addition, we have demonstrated that the severely impaired humoral response of ESRD patients to HBV vaccination can be attributed to a specific deficit in the generation of antigen-specific effector-memory CD4^+ ^T cells [9]. Therefore, the disturbed composition of circulating T cells in ESRD patients seems to underly, at least partly, their immune deficiency.

Cytomegalovirus (CMV) seropositivity is known to have a major impact on the repertoire of antigen specific T cells as an estimated 10% of circulating T cells is CMV-antigen specific in seropositive individuals [10]. In addition, the changes generally observed with an aged immune system, like a decreased CD4/CD8 ratio and expansion of CD28 negative CD8^+ ^positive T cells seem to be related to CMV seropositivity [11-13]. The prevalence of CMV seropositivity increases with age and varies with socio-economic and ethnic background from 30 to 100% [14]. Therefore, any analysis of age-related changes of the immune system should take CMV-seropositivity into account. To date few studies have documented the interaction between CMV-seropositivity and ageing on the absolute numbers of circulating T cell subsets in healthy controls [11,15,16]. Although these studies indicated an CMV-related increase in total numbers of CD8^+ ^T cells, they reached dissimilar conclusions with respect to CMV-related changes of T cells at young age which may be due to differences in definition of the various T cell populations.

ESRD patients have increased numbers of circulating CD8^+ ^Temra cells and CD28 negative T cells suggesting a major influence of CMV seropositivity on T cell immunity [17]. However, it is not known how CMV and ageing interact and contribute to the observed ESRD-related changes in numbers of circulating T cells subsets. A carefull dissection of the influence of these different factors (loss of renal function, age and CMV seropositivity) on circulating T cells is pivotal to understand the ESRD-related immune deficiency.

To examine this question, we have performed a large cross-sectional study including ESRD patients and healthy donors.

## Material and methods

### Subjects

Patients aged 18 to 78 years were recruited for the study from our outpatient clinic for renal transplantation during a period of 2.5 years. All patients had ESRD, defined as an estimated glomerular filtration rate of less then 15 mL/min with or without renal replacement therapy. Patients with a malignancy, active infection or taking immunosuppressive drugs were excluded. The healthy individuals were recruited from their relatives, thereby matching for socio-economic and ethnical background. All patients and healthy controls included gave informed consent and the local medical ethical committee approved the study. It was conducted according to the principles of Declaration of Helsinki and in compliance with International Conference on Harmonization/Good Clinical Practice regulations. Study population characteristics are depicted in Table 1. We aimed at 5 to 10 CMV-seropositive and -seronegative individuals at each age-decade.

**Table 1:** Study population characteristics

		ESRD patients	healthy controls	P-value
number of individuals		139	111	
				
age (yrs)*		46 (18-78)	50 (21-78)	0.13
				
sexe (male)		75%	48%	< 0.01
				
CMV seropositive		53%	59%	0.10
				
CMV IgG (AU/mL)*		54 (6-252)	47 (6-199)	0.54
				
underlying kidney disease				
				
	hypertensive nephropathy	36.6%		
	primary glomerulopathy	20.1%		
	diabetic nephropathy	7.5%		
	polycystic kidney disease	0.0%		
	other	30.6%		
	unknown	5.2%		
type of renal replacement therapy				
	hemodialysis	41%		
	peritoneal dialysis	24.6%		
	previous kidney transplantation	4.5%		
	none	29.9%		

* values are median (range), percentages are means.

Distribution of gender and CMV-seropositivity within the healthy donor and ESRD patients was tested using the Chi-square test. Gender did not significantly alter the composition of T lymphocyte subsets.

Comparison between the healthy donors and ESRD patients with respect to age, CMV-serology was tested using the Mann-Whitney U-test

### Flowcytometric analysis of the various T lymphocyte subsets

To determine the percentages and absolute numbers of the various T lymphocytes, we performed a whole blood staining as described previously but with some minor adjustments [7]. Briefly, to distinguish the various T lymphocyte subsets, we used AmCyan-labelled CD3 (BD, Erembodegem, Belgium) with either peridinin chlorophyll protein (PerCP)-labelled CD4 or CD8 in combination with allophycocyanin (APC)-labelled CD45RO (BD) or Pacific Blue-labelled CD45RO (Biolegend Europe B.V, Uithoorn, Netherlands), and fluorescein isothiocyanate (FITC)-labelled CCR7 (R&D Systems Europe Ltd, Abingdon, UK). The various subsets are defined as CD4^+ ^or CD8^+ ^and either naive being CCR7^+ ^CD45RO^- ^(Tnaive) or belonging to the memory population (Tmem). The latter can be further dissected in central memory being CCR7^+ ^CD45RO^+ ^(Tcm), effector memory being CCR7^-^CD45RO^+ ^(Tem) and for the CD8^+ ^T lymphocytes, Temra being CCR7^- ^CD45RO^-^. In addition, percentages of CD28^- ^(CD28null) and CD57^+ ^for CD4^+ ^and CD8^+ ^T lymphocytes were determined using phycoerythrin (PE)-labelled CD28 (BD) and APC-labelled CD57 (Biolegend), respectively. For each measurement, we acquired 50000 lymphocytes to ensure reliable results. Analysis was performed using the FACS Canto™ II (BD) and FACSDIVA software (BD).

### CMV serology

Serum IgG antibodies to CMV were measured with an enzyme immuno assay (Biomerieux, VIDAS, Lyon, France) and expressed as arbitrary units/ml (AU/mL). Following the manufacturers guidelines, a test result exceeding 6 AU/mL was considered positive for the presence of CMV-specific IgG antibodies.

### Statistical analysis

The non-parametric Spearman's rank correlation (Rs) test was performed to analyze correlations between age and the various T lymphocytes subsets in the group of healthy individuals and ESRD patients. ESRD patients and their age-matched healthy controls were divided into two groups; 20-40 years (young) and 60-80 years (old), to evaluate the effect of CMV seropositivity on the composition of the T lymphocyte compartment. The Mann-Whitney U-test was used to compare values between CMV-seronegative and seropositive individuals as well as to compare ESRD patients to healthy donors. Distribution of gender and CMV-seropositivity was tested using the Chi-square test. P-values less than 0.05 were considered significant. The SPSS software version 17 was used for all statistical tests.

## Results

### CMV-seropositivity is associated with increased T cell numbers and differentiation to a memory-effector phenotype in healthy young individuals

At young age, the absolute number of CD4^+ ^and CD8^+ ^T cells was increased in CMV-seropositive healthy individuals by 76% and 83%, respectively when compared to CMV-seronegative healthy individuals (Table 2). Both the naïve and memory T cells showed increased cell numbers, although the memory compartment contributed for the largest part (48% for CD4^+ ^and 64% for CD8^+ ^T lymphocytes). In both CD4^+ ^and CD8^+ ^T memory cells a significant increase in differentiation towards the effector-memory phenotype was observed. Within the memory compartment, the CD4 Tem increased by 2.1-fold and the CD8 Tem by 1.5-fold. The memory CD8^+ ^T cells showed the highest degree of differentiation to an effector phenotype as the Temra cells were on average 2.3-fold increased in CMV-seropositive healthy donors (Table 2).

**Table 2:** The effect of age and cytomegalovirus (CMV) serostatus on the various T lymphocytes for healthy donors

	young (20-40 yrs)			elderly (> 60 yrs)		
	CMV- (N = 15)	CMV+ (N = 17)		CMV- (N = 21)	CMV+ (N = 23)	
	mean ± SEM	mean ± SEM	P-value	mean ± SEM	mean ± SEM	P-value
T lymphocytes	0.77 ± 0.07	1.39 ± 0.13	< 0.01	0.91 ± 0.1	1.03 ± 0.09	0.19
						
CD4^+ ^T lymphocytes	0.49 ± 0.06	0.86 ± 0.09	< 0.01	0.63 ± 0.08	0.63 ± 0.06	0.74
Tnaive	0.17 ± 0.02	0.36 ± 0.05	0.01	0.25 ± 0.05	0.21 ± 0.03	0.61
Tcm	0.2 ± 0.03	0.26 ± 0.02	0.08	0.21 ± 0.02	0.23 ± 0.03	0.91
Tem	0.07 ± 0.01	0.15 ± 0.02	< 0.01	0.08 ± 0.01	0.11 ± 0.01	0.05
Tmem	0.27 ± 0.04	0.4 ± 0.04	0.02	0.29 ± 0.03	0.34 ± 0.04	0.38
*CD28null	0.11 ± 0.02	4.21 ± 0.98	< 0.01	0.34 ± 0.17	4.73 ± 1.36	< 0.01
*CD57^+^	5.23 ± 0.71	18.11 ± 3.88	0.04	6.46 ± 0.69	13.18 ± 3.77	0.34

CD8^+ ^T lymphocytes	0.23 ± 0.02	0.42 ± 0.04	< 0.01	0.22 ± 0.03	0.34 ± 0.03	< 0.01
Tnaive	0.15 ± 0.03	0.22 ± 0.04	0.28	0.06 ± 0.01	0.06 ± 0.01	0.54
Tcm	0.04 ± 0.01	0.04 ± 0.01	0.77	0.05 ± 0.01	0.08 ± 0.02	0.55
Tem	0.06 ± 0.01	0.09 ± 0.02	0.08	0.07 ± 0.01	0.11 ± 0.02	0.05
Temra	0.04 ± 0.01	0.09 ± 0.02	0.01	0.06 ± 0.02	0.11 ± 0.02	0.01
Tmem	0.14 ± 0.01	0.23 ± 0.03	0.01	0.18 ± 0.03	0.3 ± 0.03	< 0.01
*CD28null	31.89 ± 5.12	47.82 ± 4.13	0.04	27.78 ± 3.52	43.28 ± 4.47	0.02
*CD57^+^	38.39 ± 8.67	49.63 ± 6.14	0.34	29.21 ± 2.79	49.65 ± 9.38	0.03
						

CD4/CD8 ratio	2.06 ± 0.35	2.13 ± 0.18	0.571	3.58 ± 0.38	2.05 ± 0.20	< 0.01

absolute values are 10^6^/mL, Tmem is sum of Tcm+Tem or sum of Tcm+Tem+Temra for CD4 and CD8 T lymphocytes, respectively

* are percentages of CD4+ and CD8+ Tmem lymphocytes, respectively.

P-values represent the comparison between CMV- and CMV+

### CMV-seropositivity selectively influences T cell subsets with increasing age

**Figure 1:**
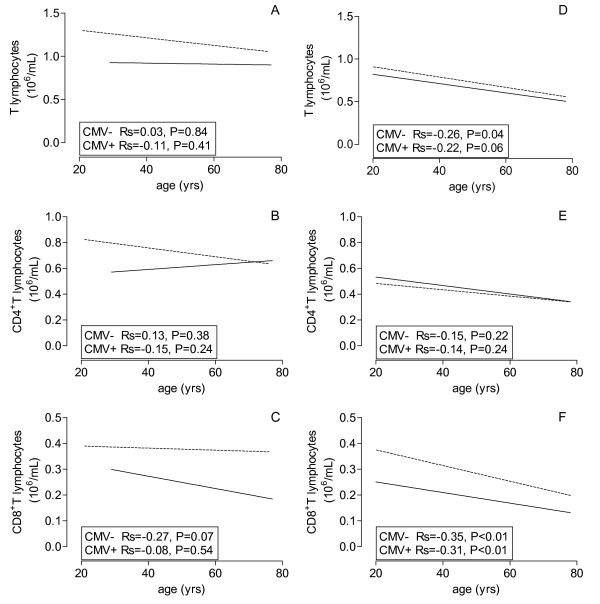
**Age-related effects of CMV on composition of T lymphocytes**. The lines of the best fits are shown for absolute numbers of T lymphocytes (A, D), CD4^+ ^(B, E) and CD8^+ ^T lymphocytes (C, F) of healthy volunteers (A, B, C) and ESRD patients (D, E, F) against age on X-axis and CMV serostatus (dotted line = CMV seropositive and closed line = CMV seronegative).

**Figure 2:**
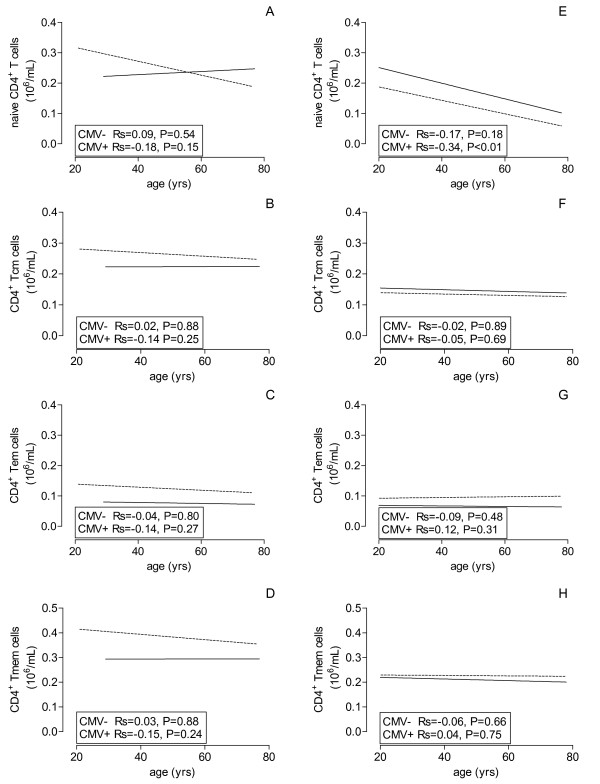
**Age-related effects of CMV on CD4^+ ^T cell subsets**. The lines of the best fits are shown for absolute numbers of CD4^+ ^Tnaive (A, E), Tcm (B, F), Tem (C, G) and Tmem (D, H) of healthy volunteers (A, B, C, D) and ESRD patients (E, F, G, H) against age on X-axis and CMV serostatus (dotted line = CMV seropositive and closed line = CMV seronegative).

With increasing age, the CD4^+ ^T lymphocyte population and the different subsets remained remarkably unaffected in CMV-seronegative persons over a period of 4 to 5 decades (Figure 1B and Figure 2A-D, straight line). However, in CMV-seropositive healthy individuals the elevated CD4 T cell numbers at young age, specifically the Tnaive, decreased to values similar to seronegative persons at old age (Table 2 Figure 1B and Figure 2A, dotted line).

**Figure 3:**
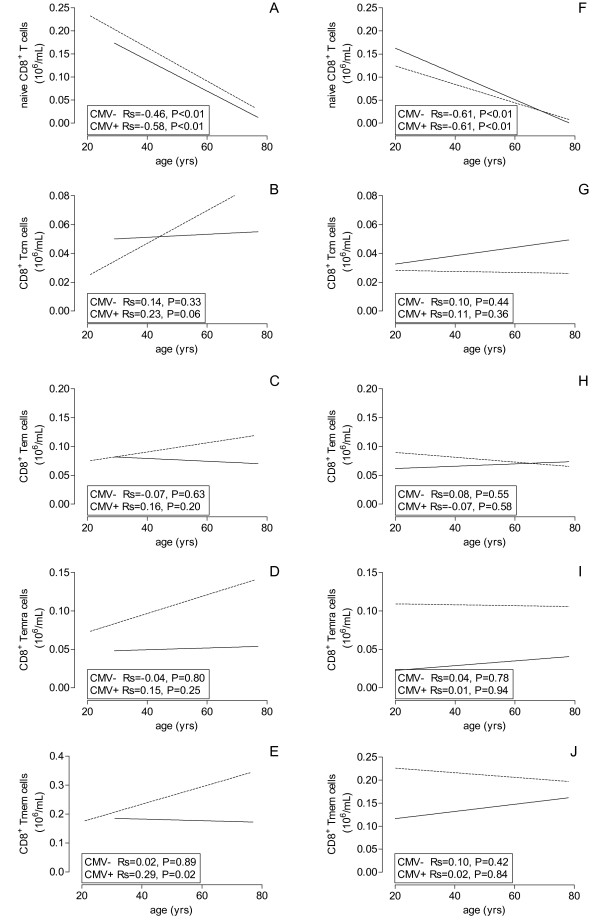
**Age-related effects of CMV on CD8^+ ^T cell subsets**. The lines of the best fits are shown for absolute numbers of CD8^+ ^Tnaive (A, F), Tcm (B, G), Tem (C, H), Temra (D, I) and Tmem (E, J) of healthy volunteers (A, B, C, D, E) and ESRD patients (F, G, H, I, J) against age on X-axis and CMV serostatus (dotted line = CMV seropositive and closed line = CMV seronegative).

The findings were very different for age-related changes of CD8^+ ^T cells. Most notably, a steep decrease (> 80%) was observed in Tnaive cell numbers which was unaffected by CMV- seropositivity. In CMV-seronegative individuals this resulted in a modest decrease of the total CD8^+ ^T cell population, as the Tmem remained unchanged with increasing age (Table 2). In CMV-seropositive individuals the CD8^+ ^Tmem increased significantly (P = 0.02) with increasing age (Figure 3E), an effect which could be observed in all memory subpopulations although statistical significance was not reached (Figure 3B-D). This resulted in a significantly age-related decrease in the CD4/CD8 ratio (P < 0.01) in CMV-seropositive healthy individuals, but not in CMV seronegative individuals (data not shown).

### Impact of CMV-seropositivity on composition of T lymphocytes subsets in ESRD patients

Compared to CMV-seropositive healthy individuals, ESRD patients have a significantly (P < 0.01) lower number of T lymphocytes which is related to a relative CD4^+ ^T cell lymphopenia at all ages. The decreased CD4^+ ^T cell numbers are caused by a significant decrease in Tnaive and Tcm as has been described before [7]. The CD4^+ ^Tnaive decreased with increasing age but this association was only statistically significant (P < 0.01) in CMV-seropositive patients (Figure 2E), and resulted in a significantly (P = 0.03) lower number of Tnaive at old age in CMV-seropositive patients (Table 3).

**Table 3:** The effect of age and cytomegalovirus (CMV) serostatus on the various T lymphocytes for ESRD patients

	young (20-40 yrs)			elderly (> 60 yrs)		
	CMV- (N = 27)	CMV+ (N = 23)		CMV- (N = 15)	CMV+ (N = 25)	
	mean ± SEM	mean ± SEM	P-value	mean ± SEM	mean ± SEM	P-value
T lymphocytes	0.72 ± 0.08	0.86 ± 0.1	0.47	0.48 ± 0.04	0.6 ± 0.04	0.09
						
CD4^+ ^T lymphocytes	0.46 ± 0.05	0.46 ± 0.06	0.90	0.35 ± 0.03	0.36 ± 0.03	0.87
Tnaive	0.2 ± 0.03	0.17 ± 0.04	0.73	0.13 ± 0.02	0.08 ± 0.01	0.03
Tcm	0.15 ± 0.02	0.14 ± 0.02	0.61	0.13 ± 0.02	0.13 ± 0.02	0.85
Tem	0.07 ± 0.01	0.09 ± 0.02	0.52	0.06 ± 0.01	0.09 ± 0.01	0.01
Tmem	0.21 ± 0.02	0.23 ± 0.03	0.93	0.18 ± 0.03	0.22 ± 0.02	0.28
*CD28null	0.05 ± 0.01	4.05 ± 0.83	< 0.01	0.82 ± 0.69	11.91 ± 2.79	< 0.01
*CD57^+^	9.93 ± 1.24	34.02 ± 7.09	< 0.01	7.61 ± 1.77	35.71 ± 7.15	< 0.01
						

CD8^+ ^T lymphocytes	0.23 ± 0.03	0.34 ± 0.04	0.02	0.17 ± 0.02	0.22 ± 0.03	< 0.01
Tnaive	0.13 ± 0.02	0.11 ± 0.01	0.64	0.03 ± 0.01	0.03 ± 0.01	0.51
Tcm	0.04 ± 0.01	0.03 ± 0.01	0.59	0.04 ± 0.01	0.03 ± 0.01	0.64
Tem	0.07 ± 0.02	0.07 ± 0.02	0.73	0.06 ± 0.01	0.06 ± 0.01	0.76
Temra	0.02 ± 0.01	0.12 ± 0.02	< 0.01	0.02 ± 0.01	0.11 ± 0.02	< 0.01
Tmem	0.13 ± 0.03	0.22 ± 0.04	0.03	0.12 ± 0.02	0.19 ± 0.03	0.02
*CD28null	26.73 ± 2.82	50.95 ± 5.55	< 0.01	24.92 ± 3.51	50.45 ± 4.44	< 0.01
*CD57^+^	26.71 ± 5.11	68.12 ± 6.7	< 0.01	34.09 ± 4.83	55.22 ± 7.26	0.06
						

CD4/CD8 ratio	2.32 ± 0.27	1.62 ± 0.18	< 0.01	4.37 ± 0.90	2.0 ± 0.20	< 0.01

absolute values are 10^6^/mL, Tmem is sum of Tcm+Tem or sum of Tcm+Tem+Temra for CD4 and CD8 T lymphocytes, respectively

* are percentages of CD4+ and CD8+ Tmem lymphocytes, respectively.

P-values represent the comparison between CMV- and CMV+

Similar as observed in healthy individuals, the CD4^+ ^T memory compartment (Figure 1E) and its subsets (Figure 2E-H) remained stable with age, irrespective of CMV-serostatus. The influence of CMV-seropositivity on differentiation to an effector memory phenotype was less clear in ESRD patients, compared to healthy individuals.

The CD8^+ ^Tnaive population showed a very significant age-related decrease, irrespective of CMV-serostatus and with a similar slope as healthy individuals. At all ages, CD8^+ ^T lymphocytes of CMV-seropositive ESRD patients are significantly higher due to an increased Tmem when compared to their seronegative counterparts similar to healthy donors. The Tmem does not increase with age (Figure 3J) as was observed for CMV-seropositive healthy individuals. At young age, CD8^+ ^Temra and Tmem are approximately 6- and 1.6-fold increased whereas at old age the values are 5.5- and 1.7-fold (Table 3). The decrease of Tnaive cell numbers but unchanged Tmem subsets led to a significant age-related decrease of total CD8^+ ^T cell numbers in ESRD patients (Figure 1F).

### CMV seropositivity and ESRD increase CD57 expression and loss of CD28 on memory T cells

Markers specific for terminal differentiation of T lymphocytes, i.e. loss of CD28 expression (CD28null) and percentages CD57^+ ^T lymphocytes are specifically higher within CD4^+ ^Tmem of CMV-seropositive healthy individuals. Similar results were obtained for percentages of CD28null, but not CD57^+^, Tmem cells within the CD8^+ ^T lymphocyte compartment. With increasing age, these markers remained stable (Table 2).

Similar to the healthy counterparts, significantly higher percentages of CD28null and CD57^+ ^in both CD4^+ ^and CD8^+ ^Tmem lymphocytes were observed in CMV-seropositive ESRD patients (Table 3). Moreover in contrast to healthy donors, a significant increase in percentages of CD28null CD4^+ ^Tmem was observed when old ESRD patients were compared to young ones (Table 3, 11.9% to 4.0%, p < 0.001). Also, the percentage of CD57 positive CD4^+ ^Tmem was significantly higher in CMV-seropositive ESRD patients as compared to healthy controls (at young age 34.0% versus 18.1% and at old age 35.7% versus 13.2% for CMV-seropositive ESRD patients and CMV-seropositive healthy donors, respectively, p < 0.01.)

## Discussion

In this study we have analyzed the differential and combined effects of age, CMV latency and loss of renal function on numbers and composition of the various T lymphocytes. The results show that CMV latency, defined by CMV seropositivity, has a major impact on circulating T cell numbers, composition and age-related effects of the immune system. Moreover, CMV aggravates some pre-existing changes in the T cell compartment of ESRD patients.

A remarkably stable composition of the CD4^+ ^T cell compartment was noted in CMV-seronegative healthy donors over an age span of at least 5 decades. Both total number of CD4^+ ^T cells and relative distribution remained unchanged in the elderly population. In contrast, the CD8^+ ^naive T cell subset showed a major age-related decline while the memory compartment remained stable with respect to differentiation and expressing of ageing markers CD28null and CD57. The preferential age-related decrease of CD8 Tnaive cells is in accordance with previous studies [18] and, although speculative, may be caused by a higher antigen driven turnover of these cells as compared to CD4^+ ^T naive cells.

CMV-seropositivity has a large effect on CD4^+ ^and CD8^+ ^T cell subsets at all ages in healthy individuals. It is associated with increased numbers of all CD4^+ ^T cells at young age and smaller numbers at old age, in particular within the Tnaive cell subset.

CMV-seropositivity was associated with a different effect on CD8^+ ^T cells, as only the memory T cell compartement was significantly and stably expanded. The slope of the age-related decline of CD8 Tnaive was not affected by CMV-seropositivity. Confirming previous results, the appearance of CD28null cells within the CD4^+ ^Tmem cell compartment seemed pathognomonic for CMV-seropositivity, but was unaffected by increasing age [15,17,19]. Overall, the results indicated that loss of CD28 expression and increased expression of CD57 on CD4^+ ^and CD8^+ ^T cells was highly associated with CMV-seropositivity. These markers should therefore be interpretated with care in clinical studies that study the ageing of T cells, taking into account the potential confounder of increasing prevalence of CMV-seropositivity with age. Hypothetical extrapolation of the regression lines of the total CD4^+ ^and CD8^+ ^T cell (sub) populations beyond 80 years results in a lowered CD4/CD8 T cell ratio, lowered Tnaive cell numbers and an increased percentage of memory T cells with a higher percentage of CD28null cells. These findings have indeed been described in the healthy very old and specifically in relation with CMV-seropositivity [11,20,21]. Therefore, it seems likely that the changes seen in the age span from 20 to 80 years continue within the ninth and tenth decade. The CMV-seropositivity related changes of the T cell immune system at old age are associated with an increased risk for mortality and therefore seem to be of clinical significance. Destabilization of atherosclerotic plaques by CD4^+^CD28null T cells [17] or immune suppressor activity of CD8^+^CD28null T cells [22,23] may explain this increased risk for mortality in elderly CMV-seropositive individuals.

The age-related decrease in the number of CD4^+ ^and CD8^+ ^Tnaive cells with CMV-seropositivity explains the reported age-related increased of memory T cells when percentages and not absolute numbers of cells are used [24]. However, studies involving human subjects that have reported absolute numbers of circulating T cell subsets in relation to age and CMV seropositivity are scarce [15,16]. As our results clearly show, latency for CMV is associated with a stable increase of memory CD8^+ ^T cells and an increase of memory CD4^+ ^T cells at young age. This is in sharp contrast to the results from the study by Chidrawar [16], which may be explained by the relative low number of young CMV-seropositive individuals in this study, but similar to data obtained by Looney et al.[15].

It is known that CMV antigen-specific T cells may be present at a high frequency within the pool of circulating CD4^+ ^and CD8^+ ^T cells [25,26], but our results are not in support of a limited immunological space [24,27] but actually show expansion of the T cell population under influence of CMV latency. The relative large CMV-related increase in total T cell numbers (> 30%) indicate a bystander effect on other T cells [10] which is in accordance with the discrepancy noted between e.g. the percentage of CMV-specific CD4^+ ^T cells and the higher percentage of CD4^+^CD28null cells [17,28].

In addition to the pleiotropic effects of CMV-seropositivity on T cell subsets in relation with age, the effects in ESRD patients are again different. ESRD patients have a remarkable contracted population of naive CD4^+ ^and CD8^+ ^T cells at all ages. The causes of this Tnaive lymphopenia are not certain but may involve reduced thymic output, lack of IL-7 and increased activation-induced apoptosis in ESRD patients [7,8]. Most strikingly, the association between CMV-seropositivity and increased expansion of CD4^+ ^T cells at young age was not observed in ESRD patients. In addition, independent of CMV-seropositivity an age-related progressive decline of an already contracted naive CD4^+ ^T cell at young age was observed. However, CMV-seropositivity showed an add-on effect on CD4^+ ^Tnaive lymphopenia and was associated with the development of terminally differentiated effector-memory T cells in both the CD4^+ ^and CD8^+ ^compartment. Similar to healthy individuals, the appearance of CD28null T cells and increased CD57 expression was highly associated with CMV seropositivity. In CMV-seropositive ESRD patients, the percentage of CD57 positive cells was significantly higher and the percentage of CD4^+^CD28null T cells increased with age. Overall, CMV-seropositivity aggravates the ESRD-related changes of the T cell immune system.

A unifying hypothesis may be that frequent CMV reactivation [29], [30,31] causes a lifetime activation of the immune system with an initial increase of CD4^+ ^T cells at young age but over the years exhaustion of replicative capacity with progressive decrease of naive T cells [32-34]. In ESRD a similar process occurs with continuous T cell activation possibly by the pro-inflammatory state of uremia and increased susceptibility for activation induced cell death. The net effect is severe Tnaive cell depletion and increased numbers of terminally differentiated CD4^+ ^and CD8^+ ^memory T cells which is aggravated by CMV reactivation. CMV reactivation is triggered by inflammatory stimuli creating the possibility that the pro-inflammatory condition of ESRD patients leads to more frequent subclinical CMV reactivation and even CMV disease [14,35].

The negative association of an CMV associated immune risk profile with survival in the very old is known and is likely also of clinical importance, but at a much earlier age, in ESRD patients. Understanding the combined effects of age, CMV seropositivity and ESRD on T cell immunity may help to identify patients at risk for a decreased vaccination response [36,37], at risk for cardiovascular events [17], and may guide patient-tailored immune suppression after kidney transplantation [38].

## Conflict of interest statement

The authors declare that they have no competing interests.

## Authors' contributions

NL designed the study, performed the experiments, statistical analyses and drafted the manuscript, EdW performed the experiments and MB designed the study and drafted the manuscript. All authors read and approved the final manuscript.
